# Cross Dimerization of Amyloid-*β* and αSynuclein Proteins in Aqueous Environment: A Molecular Dynamics Simulations Study

**DOI:** 10.1371/journal.pone.0106883

**Published:** 2014-09-11

**Authors:** Jaya C. Jose, Prathit Chatterjee, Neelanjana Sengupta

**Affiliations:** Physical Chemistry Division, CSIR-National Chemical Laboratory, Pune, Maharashtra, India; German Research School for Simulation Science, Germany

## Abstract

Self-assembly of the intrinsically unstructured proteins, amyloid beta (A*β*) and alpha synclein (αSyn), are associated with Alzheimer’s Disease, and Parkinson’s and Lewy Body Diseases, respectively. Importantly, pathological overlaps between these neurodegenerative diseases, and the possibilities of interactions between A*β* and αSyn in biological milieu emerge from several recent clinical reports and *in vitro* studies. Nevertheless, there are very few molecular level studies that have probed the nature of spontaneous interactions between these two sequentially dissimilar proteins and key characteristics of the resulting cross complexes. In this study, we have used atomistic molecular dynamics simulations to probe the possibility of cross dimerization between αSyn_1–95_ and A*β*_1–42_, and thereby gain insights into their plausible early assembly pathways in aqueous environment. Our analyses indicate a strong probability of association between the two sequences, with inter-protein attractive electrostatic interactions playing dominant roles. Principal component analysis revealed significant heterogeneity in the strength and nature of the associations in the key interaction modes. In most, the interactions of repeating Lys residues, mainly in the imperfect repeats ‘KTKEGV’ present in αSyn_1–95_ were found to be essential for cross interactions and formation of inter-protein salt bridges. Additionally, a hydrophobicity driven interaction mode devoid of salt bridges, where the non-amyloid component (NAC) region of αSyn_1–95_ came in contact with the hydrophobic core of A*β*_1–42_ was observed. The existence of such hetero complexes, and therefore hetero assembly pathways may lead to polymorphic aggregates with variations in pathological attributes. Our results provide a perspective on development of therapeutic strategies for preventing pathogenic interactions between these proteins.

## Introduction

Misfolding and aggregation of amyloidogenic proteins in the intra- or extra-cellular regions of the human brain are associated with multiple neurodegenerative diseases (ND) [1]–[6]. Although these diseases differ in their pathological attributes, the toxic transformations of the proteins are associated with similar pathways characterized initially by the formation of soluble oligomers, followed progressively by the emergence and elongation of protofibrillar and fibrillar aggregates [7]–[11]. Interestingly, recent clinical studies indicate that the symptoms associated with different ND can occur synergistically, leading to the worsening of overall prognosis [12], [13]. Recent experimental and theoretical studies have found that the abnormal cross interactions between different misfolded proteins could lead to such mixed pathologies [14]–[16].

Among different NDs, Alzheimers Disease (AD), Lewy Body Disease (LBD), and Parkinson’s Disease (PD) are the leading cause of dementia and moving disorders in the elderly. While oligomerisation and fibrillisation of A*β* has been identified as a toxic event in AD [2], progressive accumulation of αSyn has been linked to PD [3]. Recent studies suggest that αSyn may also have a crucial role to play in pathology of AD [3]. A large fraction of AD patients exhibit αSyn positive Lewy bodies associated with LBD in their brains [5], [17]. Evidences suggest that A*β* and αSyn interact directly *in vivo* and *in vitro* [14], [15], [18]. Transgenic mouse models demonstrate A*β* enhances αSyn accumulation and neuronal deficit [15]. Multi-dimentional NMR studies in membrane mimicking environment reported that the molecular interaction of αSyn with A*β*_40_ and A*β*_42_ are site-specific, and that membrane bound αSyn induced structural alterations that are more profound in A*β*_42_ compared to those in A*β*_40_ [14]. The same study also suggests that the oligomerization pathways for αSyn with A*β*_42_ and A*β*_40_ in the vicinity of cellular membranes are different [14]. Short MD simulations showed that A*β* and αSyn localized on a lipid bilayer surface are capable of forming ring-like hybrid structures that can porate the membrane [18]. Interestingly, recent kinetic study suggest that the fibrils and oligomers of A*β*_40_, A*β*_42_ and αSyn can function as seeds for promoting each other’s aggregation pathways [19].

Both A*β* and αSyn are intrinsically unstructured proteins (IUPs) whose pathological transformations are fundamentally dependent on their primary sequences. Although A*β* is an amphiphilic peptide, it has distinctive hydrophobic patches, particularly the central hydrophobic core L^17^VFFA^21^ and the C-terminal hydrophobic region A^30^–A^42^. The intra- and intermolecular interactions in these regions are known to lead to the compactification of this peptide in its monomeric state followed by its aggregation to form toxic species [8], [20]–[23]. In addition, the charged residues E^22^, D^23^, K^28^ of the A*β* peptide, that can form intra- and intermolecular salt bridges in the N-terminal fragment and at the central region play important roles in the peptide’s the pathological transformations [24]–[27]. αSyn is composed of three distinct regions; an N- terminal lipid binding domain (residues 1–60), a continuous hydrophobic domain (residues 61–95) and a highly acidic C terminal region. Among these, the hydrophobic segment is the non amyloid component (NAC) of the amyloid plaques found in AD [3]. The first two regions of αSyn is composed of six imperfect repeat sequence motifs KTKEGV, but the role of these repeats in the toxicity of the protein has not yet been understood.

We note that despite increasing evidences of overlapping pathologies of AD and PD and accelerated neurodegeneration arising from cross influences of A*β* and αSyn, there are relatively few molecular level studies that directly probe the interactions between these two dissimilar IUPs. To the best of our knowledge, molecular details of their spontaneous associations in regimes that resemble the aqueous cytoplasmic conditions remain uncharacterized. In this study, we have used microsecond scale unbiased molecular dynamics (MD) simulations to discern the early inter-molecular associations between the monomeric forms of A*β* and αSyn in aqueous environment. We mention here that interactions with surfaces can hinder the translation diffusion of proteins and affect the rates of their assembly and aggregation [28]–[30]. The initial diffusive regime has been noted to play important roles in self-assembly of amyloidogenic peptides [31]. Our simulations are performed such that restrictions on the initial diffusive regime due to surface tethering or adsorption are avoided. Our results indicate a high probability of cross-dimerization between the two sequentially dissimilar proteins leading to the formation of metastable complexes that may have the potential to further co-fibrillize. Principal component analysis revealed distinct association modes with variations in the strength and nature of inter-protein interactions, salt bridge propensities and extents of conformational disorder. The majority of cross-interactions were found to be driven electrostatically, with the Lys repeats of αSyn playing important roles in enhancing stability via inter-protein salt bridge formation. Remarkably, however, we also found the existence of an interaction mode that was predominantly stabilized via hydrophobic interactions. Our study provides evidence of marked heterogeneity in the cross interactions responsible for primary association of the two disease-associated IUPs. The data strongly suggest the existence of multiple pathways of cross-fibrillization between A*β* and αSyn, and therefore high degrees of polymorphism in the resultant cross aggregates.

## Methods

### Generation of Initial Monomer Conformations

We generated putative monomeric conformations of A*β* and *α*Syn monomers in aqueous environment by employing the accelerated molecular dynamics simulations (AMD) method with torsional boost [32] to suitably alter the predominantly helical, solution-state NMR structures of A*β* (1Z0Q) [33] and *α*Syn (2KWW) [34], available in the PDB database. The A*β* structure was experimentally reported via solution NMR studies in a 3∶7 mixture of hexafluoro-2-propanol and water, while the *α*Syn structure was reported in the micellar environment. AMD as implemented in the NAMD2.8 package [35] was used with the CHARMM all atom force field with CMAP correction [36], [37]. The theoretical details of the AMD method can be found in other reports [38]–[40]. Briefly, AMD ensures enhanced barrier crossing and sampling within shorter durations by altering the potential energy surface (*V(* ***r*** *)*) with the boost energy, *E_b_*, and the acceleration parameter, *α*. The modified potential V*(r) is given as,
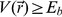
(1)

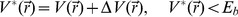
(2)


Here, the bias potential Δ*V(* ***r*** *)* is obtained as,
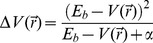
(3)


**Figure 1:**
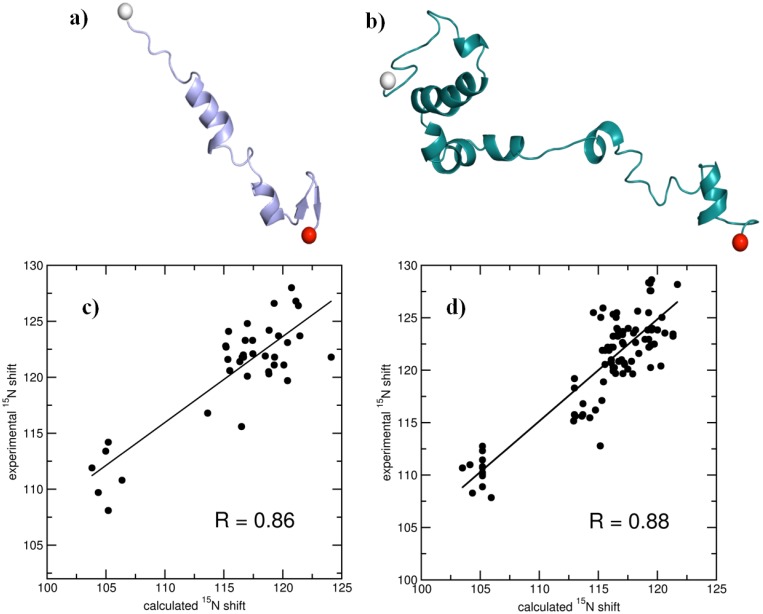
Snapshots of starting monomeric structures of a) A*β*_1–42_ and b) αSyn_1–95_ used in the unbiased simulations in the study. Correlation of average theoretical ^15^N chemical shifts with experimentally determined ^15^N chemical shifts for c) A*β*_1–42_ and d) αSyn_1–95_. The linear regressions (straight lines) and the corresponding Pearson Correlation Coefficients (R) are provided. See text for details.

Increasing values of *E_b_* and *α* result, respectively, in enhancing and reducing the extent of acceleration. In accordance with the optimized AMD methods [40] preliminary, short unbiased simulations were performed to obtain the mean dihedral energies (*V_dih_*), and *E_b_* was set such that their difference was 4 kcal mol^−1^ times the number of residues in the protein. The acidic tail region 96–140 of *α*Syn was excluded as the C-terminal truncated *α*Syn has been shown to have higher propensity for aggregation [41]–[43]. A*β* and *α*Syn are intrinsically disordered proteins with wide conformational ensembles [44]–[46]. However, the A*β* conformation obtained towards the end of our 17 ns long AMD simulations had marked similarities with important conformational members reported before, in terms of the emergence of anti-parallel C-terminal beta sheets and reduced N-terminal helicities [45], [47]. We generated an ensemble of the free peptide monomers with the conformations thus obtained, and calculated the average ^15^N chemical shift values using the SHIFTS program, [48] and compared them with the experimentally determined values for A*β* [49] and *α*Syn [50]. The mean chemical shift values were positively correlated with the experimental values. The Pearson Correlation Coefficients (PCC) for A*β* and αSyn were 0.86 and 0.88, respectively. The selected conformations, and the corresponding chemical shift correlation plots are shown in Figure 1.

### System Setup and Simulation Protocols

Spontaneous association of the A*β* and *α*Syn conformations obtained as described above were probed with unbiased simulations, also performed with the NAMD2.8 [34] package and the CHARMM force field [36], [37]. Ten independent trajectories, with the A*β* and *α*Syn placed at varying distances and relative orientations, were generated. The initial complexes were first solvated with TIP3P [51] water molecules followed by the addition of one chloride counter ion in order to neutralize the systems. We constructed large enough simulation boxes with sides extended at least 14 Å from the extremities of the proteins so that the monomers are free to interact or diffuse away. After 10,000 steps of conjugate gradient energy minimization, simulations were carried out in the isothermal-isobaric (NPT) ensemble with orthorhombic periodic boundary conditions. Constant temperature of 310 K was maintained with Langevin dynamics with a collision frequency of 1 ps^−1^, and the Langevin piston Nosé-Hoover method, was used to maintain a constant pressure of 1 atm [52], [53]. The cutoff radius for Lennard Jones interaction was set to 12 Å. SHAKE [54] was used for constraining bonds involving hydrogen atoms. Electrostatic interactions were calculated with particle-mesh Ewald method [55]. A time step of 2 fs was used. A total of 1.3 µs of unbiased simulations were generated. Pymol [56] and the VMD [57] tools were used for the generation of snapshots and visualization of the trajectories.

### Principal Component Analysis

In order to capture the most significant modes of cross-monomer interactions, clustering based on Cartesian Principal component analysis (PCA) was conducted on combined snapshots of the interacting trajectories using the program Carma [58]. PCA has been widely recognized as a reliable starting point to identify important modes of interacting systems produced by MD simulations [45], [59]–[61]. The heavily populated clusters are identified by analysing the distribution of first three principal components using an rmsd cutoff of 2.4 Å. The probability density of the distribution of first two principal components corresponding to the fluctuations of the C_α_ atoms in the bound system is calculated and converted into a free energy function using the following equation,
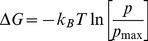
(4)where, *k*_B_ is Boltzmann’s constant, *T* is the temperature in absolute units, *p* is probability obtained from the distribution of the first two principal components, and *p*_max_ is the corresponding maximum probability.

### Configurational Entropy

We have calculated the configurational entropy per heavy atoms of A*β* and αSyn peptides in bound and unbound state using Schlitter’s method [62] as implemented in Carma program [58]. This method has been widely used to calculate the degree of change in internal conformation of bio-systems using MD trajectories [63]–[65]. Here the initial structure of each peptide is used as reference, to remove the translations and rotations with respect to the center of mass of the systems. According to Schlitter’s method the absolute entropy can be approximated as follows,
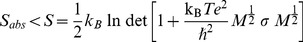
(5)Where *k*_B_ is the Boltzmann’s constant, *h-* is Planck’s constant divided by *2π*, *e* is Euler’s number, *M* is the mass matrix of *3N* dimension containing *N* atomic masses of the system and σ is the covariance matrix. The elements in the covariance matrix can be expressed as,
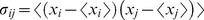
(6)where, *x_i_* and *x_j_* are the Cartesian coordinates of the selected atoms.

## Results

### I. Evaluation of inter-protein association

**Figure 2:**
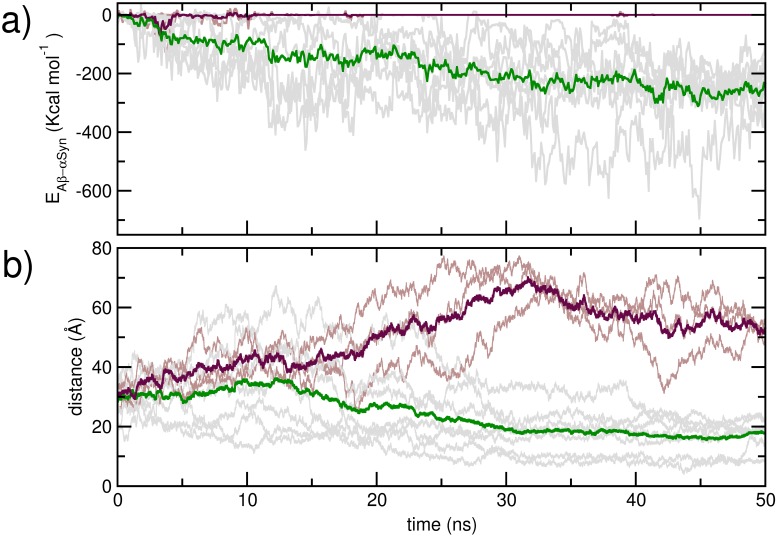
Evolution of the a) total inter-peptide interaction strength, and b) inter peptide distance over the first 50 ns of the unbiased simulation. Data for the dimerising trajectories are shown in *gray*, and averages shown in *green*; the data for non-dimerising trajectories are in *brown* and the averages shown is in *maroon.*

The initial inter-peptide center of mass distance, as well as their distances and relative orientations at 10 ns are provided in Table 1. In Figure 2 a), we present evolution of the peptide-peptide interaction strength over the first 50 ns of simulation for the trajectories. While three trajectories indicate no inter-protein interaction at the end of 50 ns, A*β* and αSyn in seven trajectories demonstrate strong interaction. The mean inter-protein interaction strength at the end of 50 ns is −172.96 (±72.8) kcal mol^−1^. We have shown corresponding evolution of the center of mass distances in Figure 2b. The mean inter-monomer center of mass distance at 50 ns of the seven trajectories where A*β* and αSyn interact are 17.5 Å, while the corresponding mean distance obtained from the non-interacting ones are 53.0 Å. The interaction energy, center of mass distances and relative orientations at 50 ns have also been provided in Table 1. The interacting trajectories were each further propagated for at least an additional 100 ns; evolutions of corresponding inter-peptide interaction strength and center of mass distances of these trajectories over 150 ns are provided in Figure S1 in File S1. We have further compared the residue-wise backbone mean squared fluctuation (MSF) in the A*β* and αSyn obtained at 0–50 ns of the simulations, with that obtained over 100–150 ns, from the peptides in the interacting trajectories (Figure S2 in File S1). An overall sharp reduction in the MSF is noted upon the formation of complexes from the two dissimilar peptides, with comparatively greater decrease in the middle regions. The interaction of A*β* and αSyn is thus commensurate with a decrease in the structural disorder.

**Table 1:** Inter-protein orientations and interaction energies along the simulation trajectories.

Traj. No.	d_0_	d_10_	θ_10_	d_50_	θ_50_	E_int_
1	33.2	31.8	79.6	12.8	101.8	−177.3
2	33.2	53.0	27.5	19.7	65.6	−173.2
3	33.2	56.8	29.8	19.1	136.3	−213.1
4	33.2	23.0	134.2	19.1	99.1	−275.0
5	33.2	38.7	135.9	55.9	60.9	0.0
6	33.2	51.7	157.8	53.2	68.5	0.0
7	18.9	13.7	162.1	21.6	109.4	−94.6
8	18.3	43.2	133.3	22.0	76.0	−403.3
9	24.3	39.4	116.9	50.1	97.4	0.0
10	24.3	26.7	119.1	8.3	158.2	−297.5

The inter-protein center of mass distances (in Å) at the start of the unbiased simulations is denoted as d_0_, at 10 ns is denoted as d_10_, and at 50 ns is denoted as d_50_. The relative orientations of the proteins are specified by the angle (in degrees) between the vectors joining the N- and C-terminii of each protein, at 10 ns (θ_10_) and at 50 ns (θ_50_). E_int_ denotes the total inter-protein interaction at 50 ns (in kcal mol^−1^).

**Figure 3:**
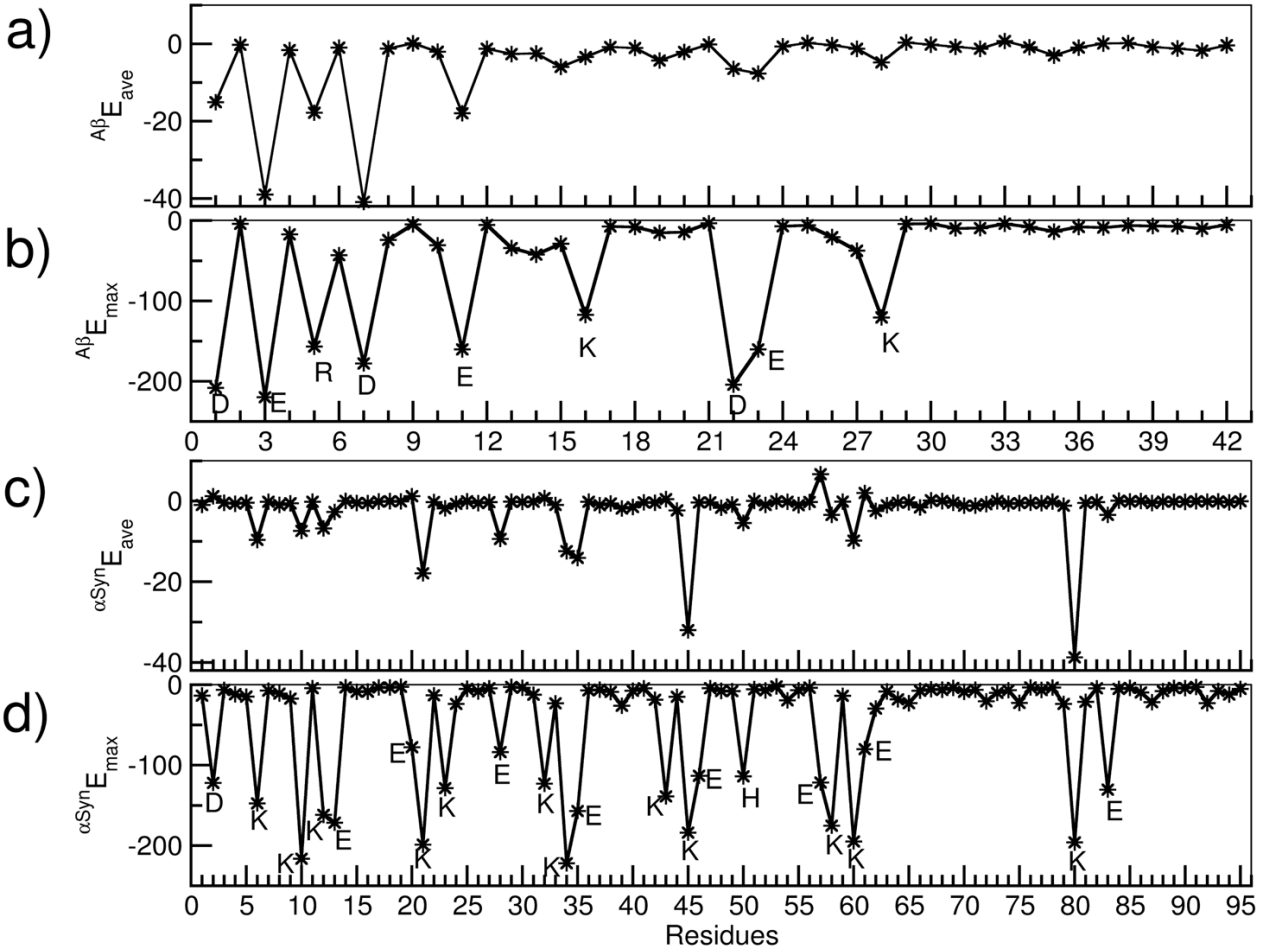
Non-bonded interaction energies in kcal mol^−1^. Residue wise: average interaction energy of A*β*_1–42_ with αSyn_1–95_ (a), maximum interaction energy of A*β*_1–42_ with αSyn_1–95_ (b), average interaction of αSyn_1–95_ with A*β*_1–42_ (c), and maximum interaction energy of αSyn_1–95_ with A*β*_1–42_ (D). The residues with strong interactions are labeled with one letter code of the respective amino acids.

The discussion above shows that despite the early diffusive regime, A*β* and αSyn have a marked, enthalpy driven propensity to interact and form dimeric complexes in aqueous solution. In Figure 3, we provide a residue-wise breakdown of the total inter-peptide interaction. Interestingly, we found that the charged residues of each peptide exhibit significantly stronger interactions compared to the hydrophobic and polar residues. This was manifestly clear when we considered the strongest interaction arising from each residue (Figure 3 b and d). Interestingly, interactions arising from the repeating Lys residues of the repeating units in the N- and C-terminii of αSyn give rise to distinctly strong interactions.

### II. Interaction heterogeneity

**Figure 4:**
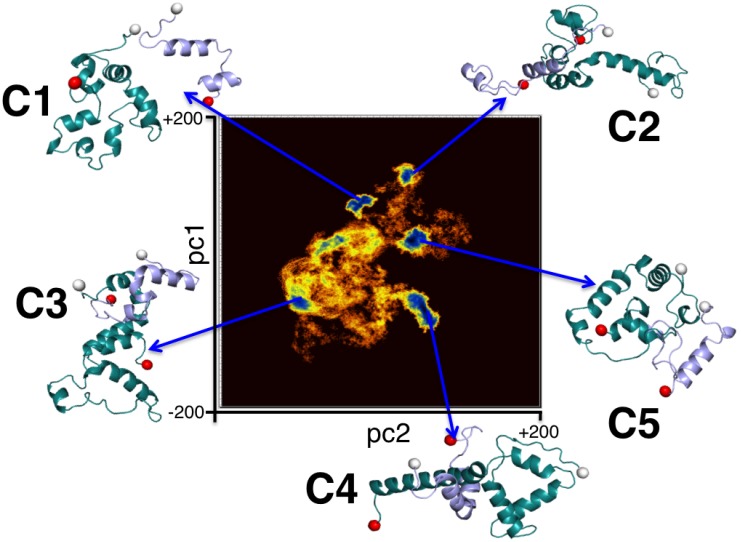
The clusters evolved during Cartesian principal component analysis (PCA) of A*β*_1–42_ and αSyn_1–95_ cross dimer system. The two-dimensional representation of the distribution of density function Δ*G,* corresponding to the fluctuations of the C_α_ atoms on the plane of the top two principal components, pc1 and pc2 is shown. The Δ*G* values spread in the range of 0 to 4.2 kcal mol ^−1^. The representative structures from five distinct clusters are shown.

Principal component analysis as described in *Methods* was performed with snapshots of the dimerized complexes where the proteins’ centers of mass were closer than 30 Å. In Figure 4, we present the free energy landscape as a function of the first (pc1) and the second (pc2) principal components. Five distinct clusters were obtained from the PCA and named C1, C2, C3, C4 and C5 in order of decreasing cluster population. Snapshots corresponding to structures residing at the cluster centers have been shown in Figure 4. In order to decipher distinguishing traits of the individual complexes in each cluster, these representative structures were individually simulated for 4 ns under the same conditions as the original simulations.

In Table 2, we have reported mean values of the number of inter-protein contacts; the radii of gyration (R_g_) of the dimeric complexes; and electrostatic and van der Waals components of the A*β*-αSyn interaction strengths of all five clusters. As in a previously reported study [20], two residues are taken to form a contact if the centers of mass of their sidechains approach within a distance of 7 Å. The five clusters are found to have significant variation in the number of inter-protein contacts, the level of compactness of the complexes as well as of the individual protein units (reported in Table S2 in File S1), and the strength of the inter-protein interactions. C5 has the highest average number of inter-protein residue contacts commensurate with the strongest inter-protein interaction (*E_tot_*) of −485.2 kcal/mol. C2 and C4 have a comparable number of inter-protein contacts, which are marginally lower than the contacts in C5. Interestingly, however, while the inter-protein interaction strength in C2 is comparable to that of C5, the interaction strength in C4 is significantly weaker, being only −82.7 kcal/mol in its mean value. Clusters C3 and C1 have markedly lower mean inter-protein contacts, with values of only 23 and 14.6, respectively. However, the inter-protein interactions in C3 and C1 are stronger than that in C4.

**Table 2:** Details of cluster heterogeneity.

Cluster	N_cont_	R_g_	E_tot_	E_Coul_	E_vdW_
C1	14.6 (4.5)	17.6 (0.4)	−146.7 (47.0)	−124.1 (48.4)	−22.6 (8.4)
C2	41.0 (3.6)	18.8 (0.6)	−361.4 (97.0)	−297.7 (96.9)	−63.8 (7.1)
C3	23.0 (4.8)	16.3 (0.3)	−158.7 (57.3)	−131.2 (55.0)	−27.6 (6.6)
C4	43.4 (3.5)	22.5 (0.7)	−82.7 (17.4)	−23.5 (16.0)	−59.2 (4.7)
C5	49.1 (5.6)	18.6 (0.5)	−485.2 (54.7)	−428.3 (55.3)	−56.9 (9.2)

The number of inter-protein contacts (N_cont_), radius of gyration of the dimer complex (R_g_), total interaction strength (E_tot_), and the electrostatic (E_coul_) and the van der Waals components (E_vdW_) of the total interaction. The units for distances and energies are Å and kcal mol^−1^, respectively.

Interestingly, we note that in C1, C2, C3 and C5, the inter-protein interaction is dominated by electrostatic energy. In contrast, in the cluster C4, the dominant non-bonded contribution arises from van der Waals interactions. However, despite the weaker inter-protein interaction, the number of residue-residue contacts in C4 is comparable to that of C5 and C2.

**Figure 5:**
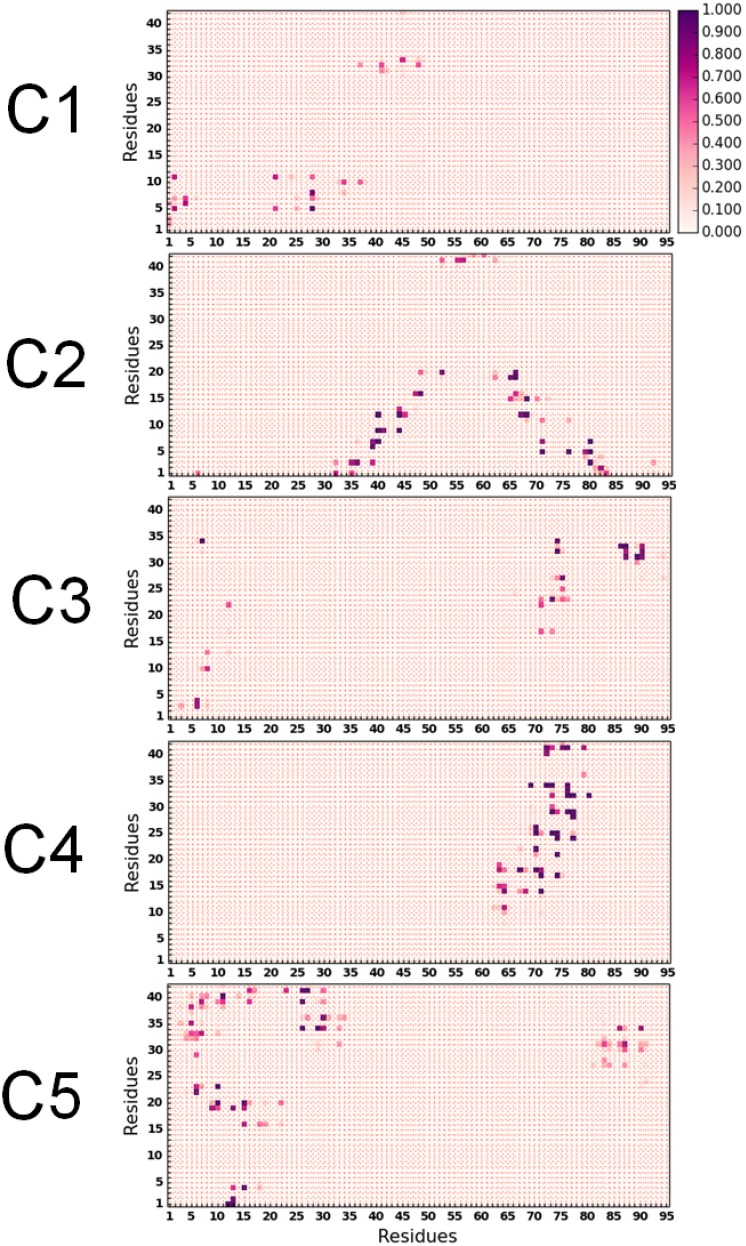
Residue specific side chain contact probability of αSyn_1–95_ with A*β*_1–42_ in different interaction sub modes.

We have compared the inter-protein side-chain contact probability maps for all five clusters in Figure 5. The contact maps reveal high degrees of contact heterogeneity amongst the various clusters. In C1, contacts were predominantly formed between the N-terminii of A*β* and αSyn. Relatively weaker contacts were noted between residues 35 to 50 of αSyn with the A*β* hydrophobic domain comprising of residues 30 to 35. In C2, the N-terminal residues of A*β* made contacts with two distinct domains of αSyn, namely the segments 32 to 53, and 63 to 85, while the A*β* C-terminal residues I^41^ and A^42^ made weaker contacts with the region A^50^–E^63^ of αSyn. C3 was predominantly characterized by N-N and C-C terminal contacts between two peptides. It is very interesting to notice that in system C4, the hydrophobic NAC region of αSyn came in close proximity of the segment 10–42 of A*β* containing hydrophobic regions 17–21, 30–35 and 39–42. In system C5, we could observe high contact probability at the terminus of both the peptides. Residues from 1–35 region of αSyn were seen to make contact with segments 1–5, 15–24 and 27–42 of A*β*. Similarly, the C-terminal residues 80–91 of αSyn made contact with the C-terminal segment 25–35 of A*β*.

We have provided inter-protein residue-residue contact energy maps corresponding to the maximum interaction strength in Figure S3 in File S1. This has been done separately for the electrostatic and the van der Waals interaction energies. We note that the strong inter-residue contact probabilities in C1, C2, C3 and C5 (as observed in Figure 5) are reflected sharply in the maximum electrostatic interactions. In contrast, the maximum contact probabilities in cluster C4 are reflected clearly in the van der Waals interactions, distinguishing this cluster from the others in the nature of interactions responsible for the dimeric complex. We note here that in every cluster except C4, the contact points were non-contiguous, and the repeating Lys residues in the αSyn sequence made significant contributions to the interaction strength.

### III. Interfacial salt bridge propensities

**Figure 6:**
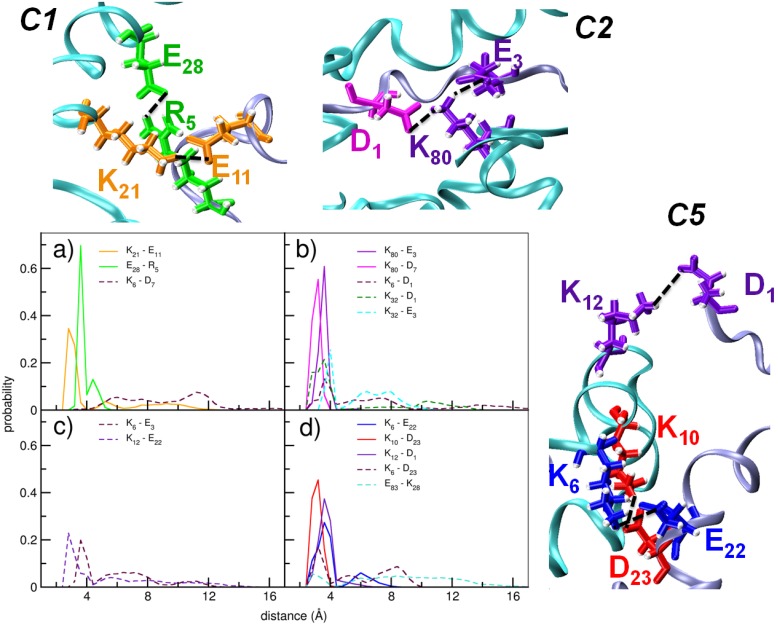
Distributions of the inter-residue distances between the residues that form inter-protein salt bridges, in clusters a) C1, b) C2, c) C3, d) C5. Distributions corresponding to the stable and the transient salt bridges are indicated in solid and broken lines, respectively. The first residue belongs to αSyn_1–95_, and the second to A*β*_1–42_. Snapshots with the stable salt bridges are shown for clusters C1, C2 and C5.

The significant electrostatic contribution to the inter-peptide interaction in the majority of clusters lead us to investigate the possible role of salt bridges in stabilizing the hybrid A*β*-αSyn complexes. We point out that inter-protein salt bridges are known to play important roles in intra- and inter-protein interactions [11], [24]–[27], [61], [66]–[69]. We utilized the VMD software for analyzing salt bridge propensities. While VMD reported no inter-peptide salt bridges in the cluster C4, multiple salt bridges were detected in clusters C1, C2, C3 and C5. In Figure 6, we present distributions of the inter-residue distances between the salt bridging pairs in C1, C2, C3 and C5. In each of these clusters, we found that the repeating Lys units of αSyn participated in all or a majority of the observed salt bridges. In C1, two salt bridges of high stability are formed between residues αSynK^21^-A*β*E^11^, and between αSynE^28^-A*β*R^5^, while a transient salt bridge is noted between αSynK^6^-A*β*D^7^. Five salt bridges were noted in C2, out of which two (αSynK^80^-A*β*E^3^ and αSynE^80^-A*β*D^7^) were highly stable, while three (αSynK^6^-A*β*D^1^, αSynK^32^-A*β*D^1^ and αSynK^32^-A*β*E^3^) were relatively more transient. The cluster C3 was found to have just two transient salt bridges, between αSynK^6^-A*β*E^3^ and αSynK^12^-A*β*E^22^. Five salt bridges were observed in cluster C5, of which the αSynK^6^-A*β*E^22^, αSynK^10^-A*β*D^23^ and αSynK^12^-A*β*D^1^ were stable and the rest (αSynK^6^-A*β*E^23^ and αSynE^83^-A*β*K^28^) transient. In Table S1 in File S1, we have reported the mean and standard deviations of the inter-residue center of mass distance between the salt bridging pairs. In clusters C2 and C5, we note the propensity to form salt bridges involving more than two charged residues. Several previous studies have highlighted important roles of such ‘complex’ salt bridges in influencing protein stabilities [70]–[72]. In C2, K32 of αSyn transiently forms salt bridges with D1 and E3 of A*β*, while K80 of αSyn forms stable salt bridges with D7 and E3 of A*β*. In C5, both K6 and K10 of αSyn are found to form salt bridges with D23 of A*β*; while the former is transient, the latter is stable. The K6 of αSyn is also noted to form a transient salt bridge with E22 of A*β*.

**Figure 7:**
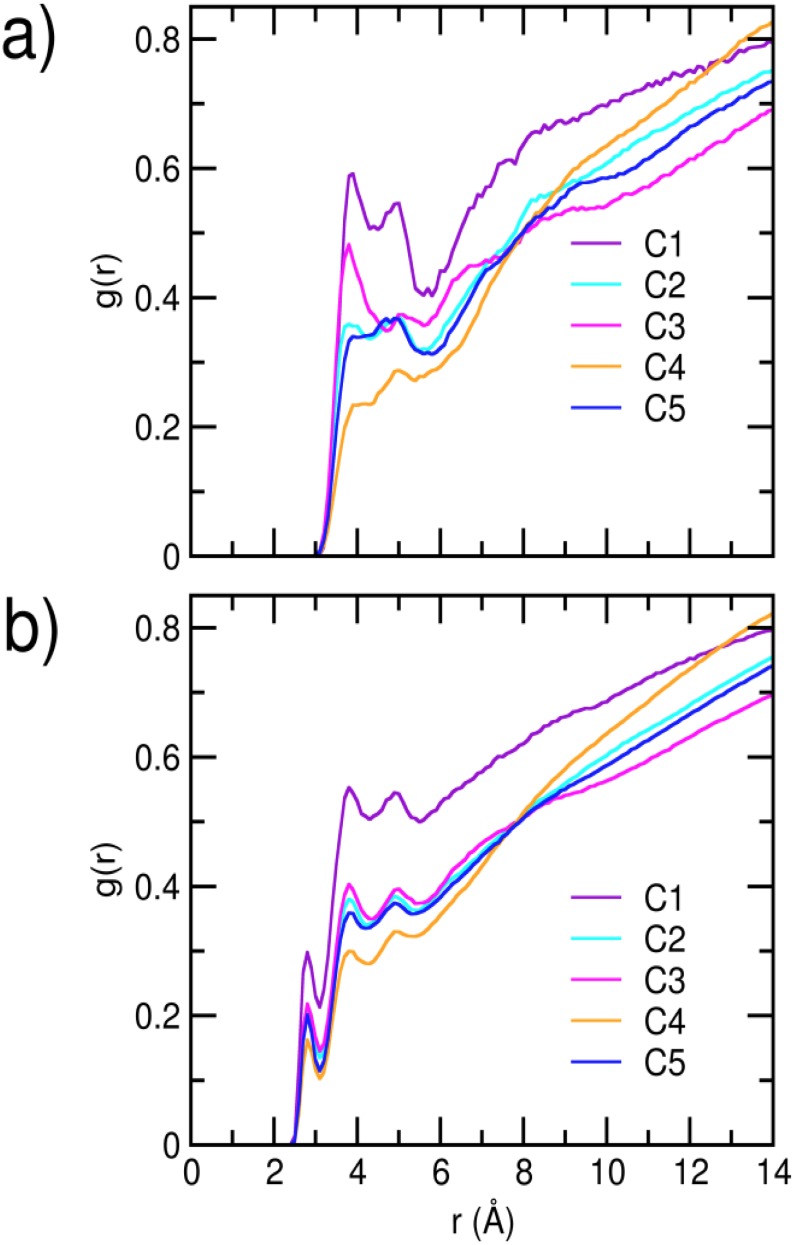
Radial distribution functions of water oxygens, around a) backbone C_α_ atoms, b) all heavy atoms of residues that make inter-protein contacts. A minimum contact probability of 0.7 has been considered.

In Figure 7, we report, for each cluster, the radial distribution functions (RDFs) calculated between oxygens of the solvent water molecules, and the C_α_ as well as the heavy atoms of residues that take display inter-residue contact. The first solvation shell of water oxygens is located at a distance of about 3.9 Å for C_α_, and at about 2.8 Å when all protein heavy atoms are considered for all clusters. For each cluster, we first note a sharp reduction in the first solvation shell of the interfacial residues compared to the full complex. Interestingly, however, the interfacial RDFs describe significant variation in the extent of hydration at the inter-protein contacts. Both C_α_ as well as the heavy atom RDFs show that the interface corresponding to cluster C4 has the least hydration, reiterating the hydrophobicity driven stability of this particular protein-protein interaction mode. Amongst the remaining clusters, we find the inter-protein interfaces of C1 and C3 to be relatively more hydrated than those of C2 and C5. It is to be noted here that salt bridge formation is often associated with a desolvation barrier [73]–[75]. Thus, the observation of a relatively drier interface in C2 and C5, compared to C1 and C3, is consistent with the observation of a greater number of interfacial salt bridges in the former clusters.

### IV. Conformational disorder

**Figure 8:**
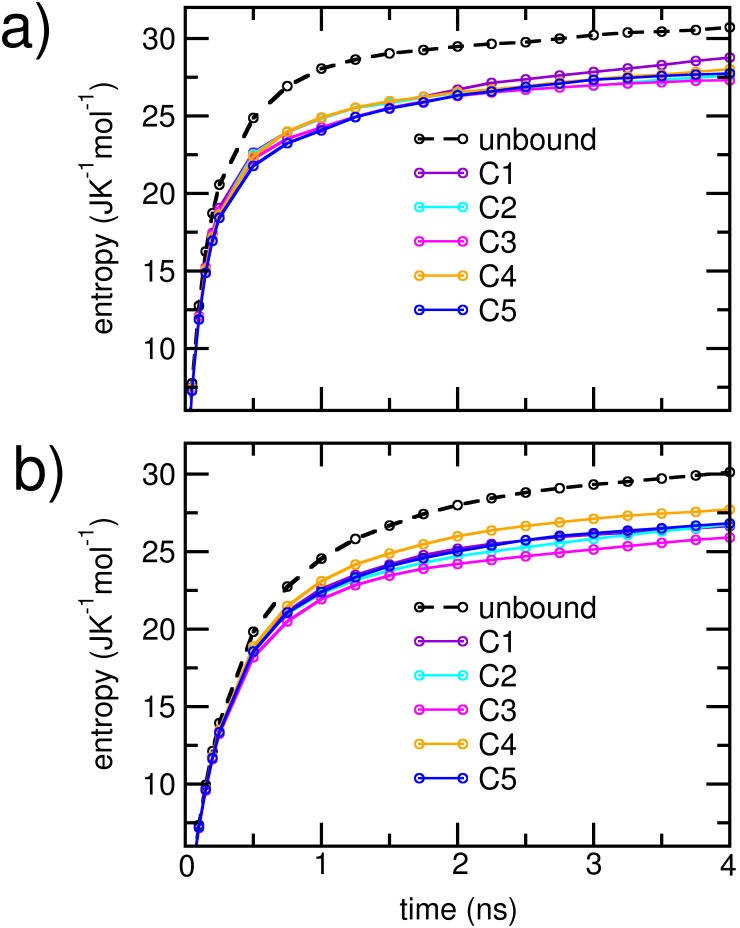
Cumulative configuration entropy per heavy atom for a) A*β*_1–42_ protein and b) αSyn_1–95_ protein. The entropy of the unbound states are denoted in black broken lines, while the entropies corresponding to the five clusters are denoted in solid, colored lines.

To compare the relative extents of disorder in each cluster, we estimated the cumulative configurational entropy per heavy atom in the individual protein units using Schlitter’s method described earlier. For comparison, we also obtained the corresponding cumulative entropies in the unbound states of the proteins. The results are plotted in Figure 8. Individual protein units in each dimerizing cluster displayed marked decrease in the net configurational entropies over the corresponding unbound state. The configurational entropies of the A*β* units had greater overlap between the clusters compared to αSyn units. In Table 3, we have listed the saturation values of the entropies and the entropy loss upon dimerization for each cluster. The configurational entropy per atom was higher for A*β*, both in the free as well as in the dimerized states. However, for a given cluster, entropy losses on the average were greater for atoms belonging to the αSyn unit. The entropy per heavy atom for αSyn and A*β* was closest in the C4 cluster, indicating the closest level of conformational disorder in the two peptides. Further, C4 was also characterized by the least overall entropy loss. However, for the clusters with stronger electrostatic interactions, we noted the absence of clear correlations between the strength of inter-protein interaction and the extent of entropy loss upon dimerization. Particularly the cluster C3, which displayed largest entropy loss, ranked third in the strength of inter-protein interactions. However, it is observed that the cluster C3 has a relatively high number of internal atom-atom contacts, particularly in the αSyn protein; this is reflected in the smaller R_g_ values (Table S2 in File S1). In comparison, the strongly associated clusters C2 and C5 had fewer internal contacts, and marginally higher configurational entropy than C3. These data suggest that the internal compactness of the protein units, particularly of αSyn, can be a contributing factor to the overall rigidity of the associated complexes.

**Table 3:** Configurational entropy calculations.

Systems	S_1_	S_2_	S_1(unbound)_–S_1_	S_2(unbound)_–S_2_	S_1_–S_2_
Unbound	30.7	30.1	-	-	0.6
C1	28.8	26.6	1.9	3.5	2.2
C2	27.6	26.8	3.1	3.3	0.8
C3	27.3	25.9	3.4	4.2	1.4
C4	28.0	27.7	2.7	2.4	0.3
C5	27.7	26.8	3.0	3.3	0.9

Configurational entropy per heavy atoms (in J K^−1^ mol^−1^) for A*β*_1–42_ (S_1_) and the αSyn_1–95_ (S_2_) proteins in the unbound states and in the clusters C1, C2, C3, C4 and C5. The entropy differences between the unbound and bound states, as well as the difference between the entropies of A*β*_1–42_ and αSyn_1–95_ are also provided.

## Discussion and Conclusion

Recent *in vitro* and *in vivo* studies report that cross interactions between dissimilar IUPs can play significant roles in clinically observed mixed pathological traits in ND patients [14]–[19], [76]–[80]. Notably, significant experimental evidence exists to suggest that A*β*, whose assembly can trigger AD, and αSyn, whose assembly is responsible for PD, can co-associate in biological milieu [14], [15], [17], [18], [76], [78], [79]. However, to the best of our knowledge, there exist no molecular level studies probing their unrestricted associations in aqueous environments. In this study, we reported the heterogeneous interactions of A*β*_1–42_ and αSyn_1–95_ from a large ensemble of the dimeric complex obtained from unbiased MD simulations of the protein sequences in explicit water.

In four out of the five interaction modes discerned with Principal component analysis, electrostatic forces are seen to dominate over van der Waals interactions. Residue specific investigations revealed the importance of the Lys residues, especially those in the imperfect repeating units of αSyn, during cross dimerisation. We note here that Lys specific molecular tweezers have been reported to be capable of inhibiting the aggregation of various amyloidogenic peptides [81]–[84]. 1,4-napthoquinone based inhibitors were also found to interact with Lys residues and efficiently reduced the fibrilisation propensity of αSyn [85]. Thus, our observation of the importance of the Lys repeats in the cross dimerization may be used for designing drugs targeted at inhibiting A*β*-αSyn co-assembly.

Clusters with dominant electrostatic interactions were characterized by the presence of multiple inter-protein salt bridges. Interestingly, the majority of salt bridges were formed between Lys residues of αSyn and Asp or Glu of A*β.* Studies suggest that the disruption of salt bridges is likely to affect the structure, toxicity and oligomerisation of αSyn [11]. Similarly, in A*β* aggregates, the salt bridge between D23 and K28 is crucial for stability of the hairpin form and formation of fibrillar aggregates [17], [24]–[27]. Further studies would reveal if the inter-protein salt bridges have any disruptive effects on the ones crucial for self-assembly, and the extent to which this may result in structural dissimilarities between the self-aggregates and the co-aggregates.

Importantly, hydrophobic interactions were also found to play crucial roles in the hetero dimerisation process. In a single interaction mode devoid of inter-protein salt bridges, the van der Waals interactions dominated over the average electrostatic interactions. In this particular system, the hydrophobic core regions comprising of 17–21, 30–35 and 39–42 of A*β* were found to be in contact with the NAC of αSyn. Additionally, we observed inter peptide contact of A*β* with residues of the NAC in all the electrostatically stabilized clusters except C1. We point out that the hydrophobic core regions in A*β* play crucial roles in its early dynamics, oligomerization and fibril formation [8], [20]–[22], [86], [87]. Similarly, in αSyn the central hydrophobic NAC region is necessary for its aggregation and this fragment is clinically observed in amyloid plaques found in patients with LBD [3], [5], [14], [88]–[91]. Earlier solid phase binding studies as well as NMR studies indicate that the NAC interacts with A*β,* particularly with residues G67, G73 and V74 and proposed a mechanism for the overlapping pathogenesis that the cleavage of NAC is catalyzed by A*β* oligomer [14]. It is worthwhile to mention here that a major strategy in the drug design against for amyloidogenic peptides is to target regions that drive hydrophobic interactions [86], [91]–[93]. Thus, the results of our analyses demonstrating NAC interaction with A*β* hydrophobic regions, along with the experimental reports, indicate that these regions could represent other plausible therapeutic targets.

Before concluding, we note that it is important to study secondary structural details of the peptide monomers during hetero assembly, and this requires careful comparison of results obtained from multiple force fields with experimental data. The clear evidence of complex formation without the emergence of strand motifs indicates that the complexes are metastable and could be prone to further assembly. Longer, millisecond timescale simulations may reveal more modes of A*β*-αSyn associations. Nevertheless, the evidence of significant heterogeneity in the nature of interactions leading to cross dimerization revealed by our microsecond simulations is strongly suggestive of heterogeneity during the seeding phase and along the early assembly pathways. This may result in the emergence of hetero oligomers and thus significant levels of polymorphism in higher ordered aggregates. In further studies, the interactions of preformed hybrid systems with lipid bilayers would greatly facilitate identification of the level of toxicity of each species. This information, along with the specific inter-residue interactions, could significantly aid the development of therapeutics against synergistic ND.

## Supporting Information

File S1 **Supporting files. Figure S1, Evolution of the a) total inter-peptide interaction strength, and b) inter peptide distance over 150 ns for the dimerising trajectories. Figure S2, The backbone mean square fluctuation (MSF) for the a) N-terminal residues, b) middle regions, and c) C-terminal residues of A** ***β*** **_1–42,_ and the d) N-terminal residues, e) middle regions, and f) C-terminal residues of αSyn_1–95_.** The data for the last 50 ns of the dimerising trajectories are shown in *gray*, with the averages in *green* (solid line). Corresponding average data for the same systems for the initial 50 ns is provided in green (broken line). Average data for the non-dimerising systems is shown in *maroon* (broken line) for comparison. **Figure S3, Residue wise maximum electrostatic (left column) and van der Waal (right column) interaction energies (in kcal mol^−1^) of αSyn_1–95_ with A** ***β*** **_1–42_ for clusters C1, C2, C3, C4 and C5. Table S1, Mean value of the inter-residue sidechain distances (d^SB^, in Å) between the residues that form salt bridges in the clusters a) C1, b) C2, c) C3, d) C5.** Standard deviations are provided in braces. The first residue belongs to αSyn_1–95_; the second residue belongs to A*β*_1–42_. **Table S2, Mean values of the total number of internal contacts formed in the A** ***β*** **_1–42_ (N_int_^Aβ^) and αSyn_1–95_ (N_int_^αS^) proteins in the five clusters.** The corresponding radii of gyration (in Å) have been denoted as R_g_^Aβ^ and R_g_^αS^.
(PDF)
