# Supplementary material for: Cross Dimerization of Amyloid-β and αSynuclein Proteins in Aqueous Environment: A Molecular Dynamics Simulations Study
Source: PLoS One. 2014 Sep 11;9(9):e106883. doi: 10.1371/journal.pone.0106883 (PMC4161357; doi:10.1371/journal.pone.0106883)
Supplement: File S1 — Supporting files. Figure S1, Evolution of the a) total inter-peptide interaction strength, and b) inter peptide distance over 150 ns for the dimerising trajectories. Figure S2, The backbone mean square fluctuation (MSF) for the a) N-terminal residues, b) middle regions, and c) C-terminal residues of A β 1–42, and the d) N-terminal residues, e) middle regions, and f) C-terminal residues of αSyn1–95. The data for the last 50 ns of the dimerising trajectories are shown in gray, with the averages in green (solid line). Corresponding average data for the same systems for the initial 50 ns is provided in green (broken line). Average data for the non-dimerising systems is shown in maroon (broken line) for comparison. Figure S3, Residue wise maximum electrostatic (left column) and van der Waal (right column) interaction energies (in kcal mol−1) of αSyn1–95 with A β 1–42 for clusters C1, C2, C3, C4 and C5. Table S1, Mean value of the inter-residue sidechain distances (dSB, in Å) between the residues that form salt bridges in the clusters a) C1, b) C2, c) C3, d) C5. Standard deviations are provided in braces. The first residue belongs to αSyn1–95; the second residue belongs to Aβ 1–42. Table S2, Mean values of the total number of internal contacts formed in the A β 1–42 (NintAβ) and αSyn1–95 (NintαS) proteins in the five clusters. The corresponding radii of gyration (in Å) have been denoted as Rg Aβ and Rg αS. (PDF) [file pone.0106883.s001.pdf]

**Supporting Information to**

# "Cross Dimerization of Amyloid-$\beta$ and $\alpha$Synuclein Proteins in Aqueous Environment: a Molecular Dynamics Simulations Study"

*Jaya C. Jose,*[†] *Prathit Chatterjee*[†] *and Neelanjana Sengupta*[†*]

[†]*Physical Chemistry Division*

*CSIR-National Chemical Laboratory,*

*Pune, Maharashtra,*

*India*

[*] Corresponding author: *E-mail address*: n.sengupta@ncl.res.in

Phone: +91-20-25902087

Fax: +91-20-25902636

**Table S1.** Salt bridge distances

| Cluster | Salt Bridge | $d^{SB}$ |
|---|---|---|
| C1 | K6 – D7 | 9.1 (2.9) |
| | K21 – E11 | 4.5 (2.7) |
| | E28 – R5 | 3.6 (0.5) |
| C2 | K6 – D1 | 7.4 (4.3) |
| | K32 – D1 | 5.5 (3.4) |
| | K32 – E3 | 5.9 (1.9) |
| | K80 – D7 | 2.9 (0.2) |
| | K80 – E3 | 3.3 (0.3) |
| C3 | K6 – E3 | 5.9 (2.4) |
| | K12 – E22 | 5.7 (3.2) |
| C5 | K6 – E22 | 4.0 (1.3) |
| | K6 – D23 | 5.6 (2.2) |
| | K10 – D23 | 2.9 (0.3) |
| | K12 – D1 | 3.5 (0.6) |
| | E83 – K28 | 8.5 (3.4) |

Mean value of the inter-residue sidechain distances ($d^{SB}$, in Å) between the residues that form salt bridges in the clusters a) C1, b) C2, c) C3, d) C5. Standard deviations are provided in braces. The first residue belongs to $\alpha Syn_{1\text{-}95}$; the second residue belongs to $A\beta_{1\text{-}42}$.

**Table S2.** Details of internal contacts and compactness of peptides in different clusters

| **Clusters** | $<\mathbf{Nint.}^{A\beta}>$ | $<\mathbf{Nint.}^{\alpha S}>$ | $\mathbf{R_g^{A\beta}}$ | $\mathbf{R_g^{aS}}$ |
|---|---|---|---|---|
| C1 | 141.4<br>(7.4) | 485.0<br>(18.0) | 12.0<br>(0.5) | 14.6<br>(0.2) |
| C2 | 141.5<br>(9.1) | 414.6<br>(12.9) | 14.67<br>(0.7) | 16.7<br>(0.5) |
| C3 | 153.1<br>(7.6) | 467.1<br>(12.9) | 10.9<br>(0.2) | 14.4<br>(0.2) |
| C4 | 126.0<br>(5.7) | 361.9<br>(9.3) | 14.2<br>(0.4) | 23.8<br>(0.7) |
| C5 | 141.1<br>(7.8) | 454.2<br>(13.2) | 12.9<br>(0.3) | 16.9<br>(0.3) |

Mean values of the total number of internal contacts formed in the $A\beta_{1\text{-}42}$ ($N_{int}^{A\beta}$) and $\alpha Syn_{1\text{-}95}$ ($N_{int}^{\alpha S}$) proteins in the five clusters. The corresponding radii of gyration (in Å) have been denoted as $R_g^{A\beta}$ and $R_g^{\alpha S}$.

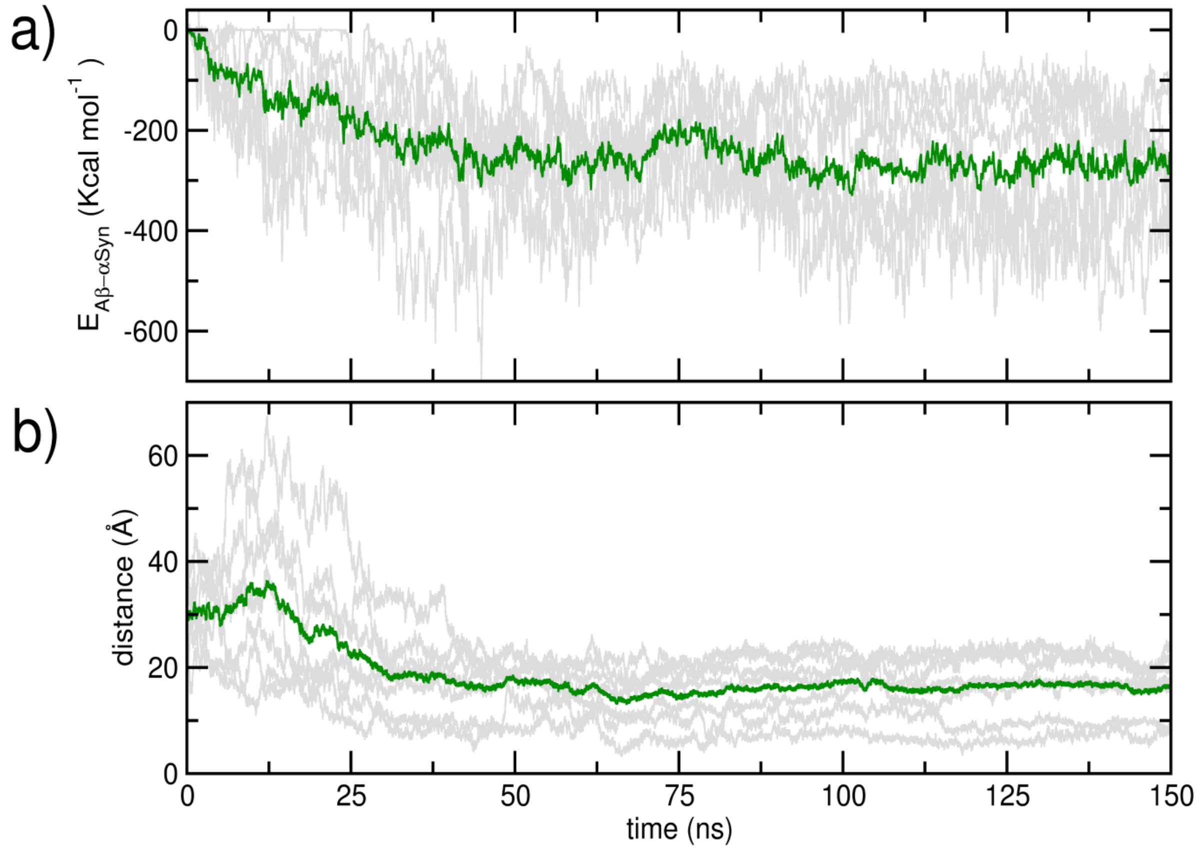


**Figure S1.** Evolution of the a) total inter-peptide interaction strength, and b) inter peptide distance over 150 ns for the dimerising trajectories.

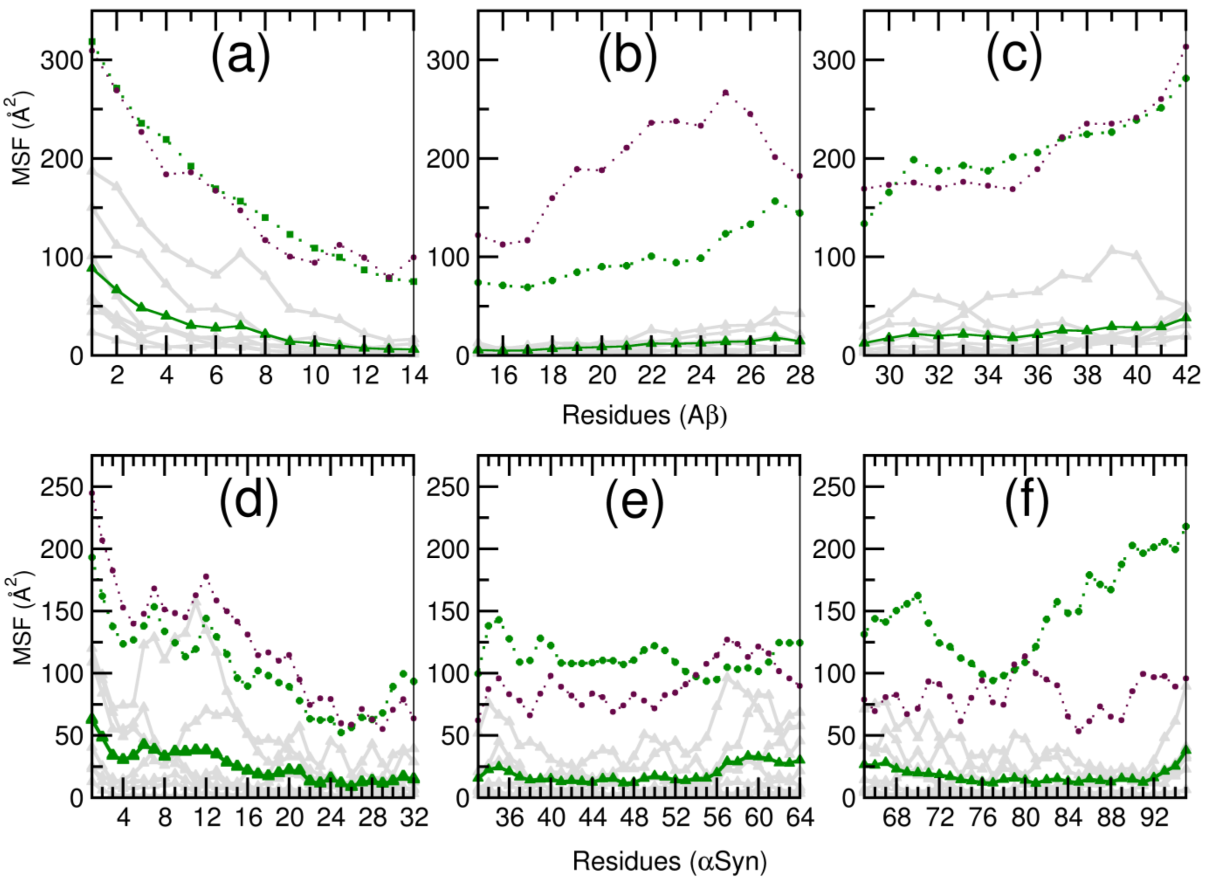


**Figure S2.** The backbone mean square fluctuation (MSF) for the a) N-terminal residues, b) middle regions, and c) C-terminal residues of $A\beta_{1\text{-}42}$, and the d) N-terminal residues, e) middle regions, and f) C-terminal residues of $\alpha Syn_{1\text{-}95}$. The data for the last 50 ns of the dimerising trajectories are shown in *gray*, with the averages in *green* (solid line). Corresponding average data for the same systems for the initial 50 ns is provided in green (broken line). Average data for the non-dimerising systems is shown in *maroon* (broken line) for comparison.

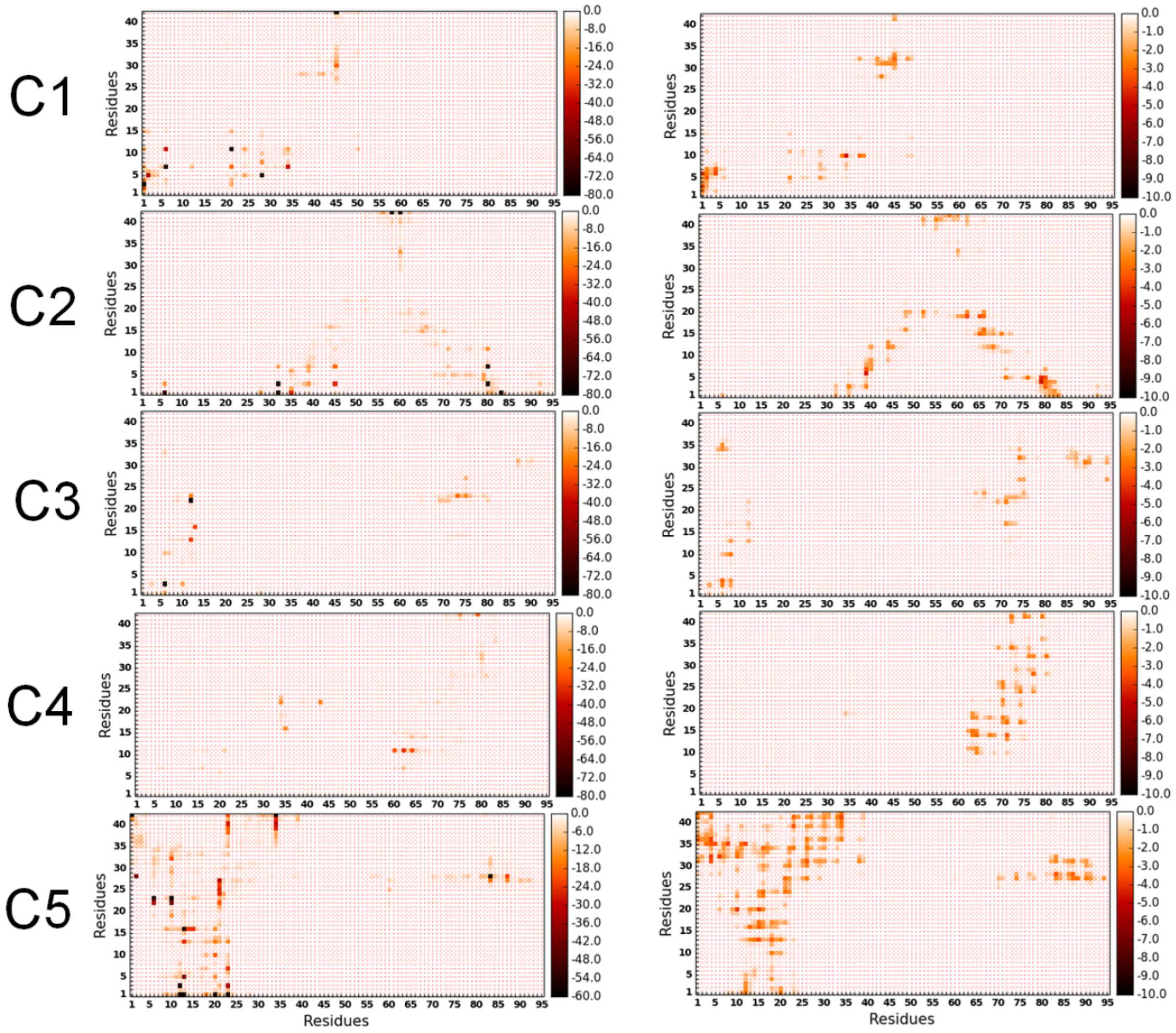


**Figure S3.** Residue wise maximum electrostatic (left column) and van der Waal (right column) interaction energies (in kcal mol$^{-1}$) of αSyn$_{1\text{-}95}$ with A$\beta_{1\text{-}42}$ for clusters C1, C2, C3, C4 and C5.
